# Comparison of Zebrafish Larvae and hiPSC Cardiomyocytes for Predicting Drug-Induced Cardiotoxicity in Humans

**DOI:** 10.1093/toxsci/kfz165

**Published:** 2019-07-30

**Authors:** Sylvia Dyballa, Rafael Miñana, Maria Rubio-Brotons, Carles Cornet, Tiziana Pederzani, Georgia Escaramis, Ricard Garcia-Serna, Jordi Mestres, Javier Terriente

**Affiliations:** 1 ZeClinics SL, IGTP (German Trias and Pujol Institute), Badalona 08916, Spain; 2 CIBER Epidemiology and Public Health; 3 Department of Biomedicine, Faculty of Life Science and Health, University of Barcelona 08036, Barcelona, Spain; 4 Research Group on Statistics, Econometrics and Health (GRECS), UdG, Girona 17071, Spain; 5 Chemotargets SL, Parc Científic de Barcelona, Barcelona 08028, Spain; 6 Systems Pharmacology, Research Program on Biomedical Informatics (GRIB), IMIM Hospital del Mar Medical Research Institute, Barcelona 08002, Spain; 7 University Pompeu Fabra, PRBB (Barcelona Biomedical Research Park), Barcelona 08002, Spain

**Keywords:** cardiovascular toxicity, adverse effect, high-throughput, drug screening, zebrafish, hiPSC, ZeCardio

## Abstract

Cardiovascular drug toxicity is responsible for 17% of drug withdrawals in clinical phases, half of post-marketed drug withdrawals and remains an important adverse effect of several marketed drugs. Early assessment of drug-induced cardiovascular toxicity is mandatory and typically done in cellular systems and mammals. Current *in vitro* screening methods allow high-throughput but are biologically reductionist. The use of mammal models, which allow a better translatability for predicting clinical outputs, is low-throughput, highly expensive, and ethically controversial. Given the analogies between the human and the zebrafish cardiovascular systems, we propose the use of zebrafish larvae during early drug discovery phases as a balanced model between biological translatability and screening throughput for addressing potential liabilities. To this end, we have developed a high-throughput screening platform that enables fully automatized *in vivo* image acquisition and analysis to extract a plethora of relevant cardiovascular parameters: heart rate, arrhythmia, AV blockage, ejection fraction, and blood flow, among others. We have used this platform to address the predictive power of zebrafish larvae for detecting potential cardiovascular liabilities in humans. We tested a chemical library of 92 compounds with known clinical cardiotoxicity profiles. The cross-comparison with clinical data and data acquired from human induced pluripotent stem cell cardiomyocytes calcium imaging showed that zebrafish larvae allow a more reliable prediction of cardiotoxicity than cellular systems. Interestingly, our analysis with zebrafish yields similar predictive performance as previous validation meta-studies performed with dogs, the standard regulatory preclinical model for predicting cardiotoxic liabilities prior to clinical phases.

Adverse effects from drugs represent a heavy burden for the healthcare sector. Among the different types of drug-induced toxicities, cardiovascular toxicity is responsible for 17.4% of clinical stage withdrawals (Fung *et al.*, 2001), and 46% of post-marketed drug withdrawals (Stevens and Baker, 2009). Those figures imply that current assessment methods for analyzing cardiotoxic liabilities promoted by drugs are insufficient and lack reliable predictive potential. Cardiotoxic liabilities endanger patients’ lives and pose a burden to the healthcare sector. They also have a negative impact on R&D economics, given the wasted expenditure in products that either failed to reach the market or were withdrawn after commercialization. Considering this health and economic impact, the pharmaceutical industry has implemented more stringent cardiovascular toxicity testings during early drug development stages (Blomme and Will, 2016). In line with this, the International Conference on Harmonization (ICH) enforced more strict guidelines for evaluating cardiotoxicity and other preclinical safety liabilities *in vitro, ex vivo*, and *in vivo.* The best example is the Guideline S7B “The Non-clinical Evaluation Of The Potential For Delayed Ventricular Repolarization (Qt Interval Prolongation) By Human Pharmaceuticals” (ICH Expert Working Group, 2005).

During early phases of drug development, *in vitro* systems such as human induced pluripotent stem cell cardiomyocytes (hiPSC-CMs) are the traditional model for high-throughput screens (HTS) of chemical libraries. As human derived cells, hiPSC-CMs retain full genetic conservation of cardiac drug targets. Hence, hiPSC-CMs can provide insights into altered cellular processes such as calcium transport, mitochondrial regulation, or ion channels function (Li *et al.*, 2016). Despite these relevant features, cellular systems do not recapitulate the structural complexity of the heart or its interplay with the vasculature. Moreover, *in vitro* models are isolated from other organs and the metabolism and, thus, cannot address the cardiotoxic impact of drug metabolites. Although hiPSC-CMs might provide a good translatability of on-target effects, their reductionist nature might reduce their predictive power when it comes to cardiotoxic adverse effects.

In later phases of drug development, large mammalian models, mainly dogs, remain the gold standard for cardiotoxicity testing (Blomme and Will, 2016). Preclinical testing in those large mammals is crucial and compulsory by regulatory agencies to grant access to clinical phases. Although these tests might be highly informative, ethical, and economic reasons make them low-throughput and extremely expensive. It is customary to use them only for testing the best preclinical lead candidate. The use of small rodents, such as mice and rats, provides a slightly higher throughput than dogs, but their physiological differences with humans generate doubts about their predictive performance (Blomme and Will, 2016). In addition to those concerns, the use of mammals for cardiotoxicity testing impacts negatively on the industry efforts for implementing animal research replacement, reduction, and refinement (3R) measures.

Aforementioned limitations of cellular and mammalian models reveal the need for alternative screening methods to streamline the prediction of drug-induced cardiotoxicity in early/mid-stage drug development phases. Along these lines, the adoption of zebrafish larvae can become a major asset for selecting safer candidates for preclinical phases and to avoid late drug attrition. Two reasons make the zebrafish an attractive model for pharmaceutical research: (1) The zebrafish genome is highly conserved (Howe *et al.*, 2013), which results in most drug metabolism components and on-/off-drug targets being conserved with humans (Peterson and MacRae, 2012). Additionally, zebrafish genetic manipulation is now easily accessible through the CRISPR technology (Cornet *et al.*, 2018). This allows interrogating the mechanism of action (MoA) and mechanism of toxicity of candidate drugs. (2) The zebrafish model is well suited for HTS approaches: zebrafish larvae are small and the large number of progeny develops externally. Moreover, tissue transparency and rapid life cycle of the larvae make them an ideal model for noninvasive image-based *in vivo* assessment of organs, and the availability of transgenic lines facilitates observing specific organs by fluorescent microscopy. Finally, zebrafish larvae younger than 5 days post fertilization (dpf) are considered *in vitro* systems by animal welfare regulation within the European Union, allowing 3R implementation by the pharmaceutical industry.

Research on the cardiovascular system can benefit from zebrafish larvae due to the following characteristics: electrophysiological maturation and beating rhythm of the zebrafish larval heart are stabilized from 96 h post fertilization (hpf) (Tessadori *et al.*, 2012). The ECG pattern includes a P-wave, a QRS-complex, and a T-wave which suggests that depolarization and repolarization kinetics in the zebrafish heart are similar to humans (Kithcart and MacRae, 2017). In fact, the zebrafish larvae heart rate of approximately 200 bpm is much closer to the human heart rate of approximately 80 bpm than is the rodent heart rate (approximately 400 bpm) (Wilkinson *et al.*, 2014). As indicated above, several fluorescent transgenic lines labeling heart (*cmlc2*: *GFP*) (Huang *et al.*, 2003), vasculature (*fli: GFP*) (Lawson and Weinstein, 2002), and blood cells (*gata1*: DsRed) (Traver *et al.*, 2003) are available to facilitate the assessment of cardiovascular function *in vivo*. Altogether, these advantages have placed zebrafish as a promising model for predicting cardiotoxicity, validating cardiovascular disease-associated genes, and discovering new therapies against human cardiovascular diseases (Asnani and Peterson, 2014). In fact, previous validation studies addressed how drug-induced cardiotoxicity in zebrafish larvae could be used to predict cardiotoxicity in humans. One study showed how 22 of 23 molecules promoting repolarization events in humans triggered the same effects in zebrafish (Milan *et al.*, 2003). Another study showed a 100% true positive rate (TPR; sensibility) for 8 cardiotoxic drugs in zebrafish (Zhu *et al.*, 2014). Recently, we published the analysis of 25 compounds, in which we obtained a prediction of human cardiotoxicity of TPR of 68% and true negative rate (TNR; specificity) of 89% (Cornet *et al.*, 2017). However, most of these studies used low-resolution imaging methods, which only allowed quantifying heart rate frequency differences.

To further validate the zebrafish larva as a suitable model for uncovering cardiotoxic liabilities we have developed ZeCardio, an integrated hardware and custom software screening platform designed for high-throughput *in vivo* imaging acquisition and analysis of complex zebrafish heart and blood flow phenotypes. We have tested the platform on a library of 92 compounds with known molecular targets and cardiotoxic activity in humans. Our study aims to provide a comprehensive comparison of drug-induced cardiotoxicity in humans with data from hiPSC-CMs and zebrafish larvae. The insights from this cross-validation study should delineate the potentials and limitations of using zebrafish larvae for detecting clinically relevant cardiotoxic liabilities and will help to determine how to position the zebrafish larva model along the drug development pipeline.

## MATERIALS AND METHODS

### 

#### Compound Library and Disproportionality Analysis

Classification of cardiotoxic versus noncardiotoxic compounds is a fundamental step toward understanding predictive performance. To that end, a disproportionality analysis, to classify those compounds into human positive (with considerable cardiotoxic liabilities in humans) and human negatives (without considerable cardiotoxic liabilities in humans), was performed on clinical data of these compounds retrieved from the FDA Adverse Event Reporting System (FAERS) database. FAERS is a collection of spontaneous reports of safety events linked to drugs made publicly available by the U.S. Food and Drug Administration (FDA). Directly downloadable from the FDA website, it currently contains 11.1 million reports from 2004 to 2018. Each report in FAERS contains data on the main drug believed to be responsible of the safety event(s) collected (primary suspect), the list of concomitant drugs coadministered to the patient, the list of safety events suffered by the patient, the therapeutic indication(s) for which the drugs were prescribed, the administration route, the gender, age, and weight of the patient, the clinical output, the actual reporter, and the event and deposit dates. These raw data went then through a careful data acceptance and curation process. First of all, duplicate reports having exactly the same data contents in all fields but deposited within a few days apart from each other were removed. Then, reports were checked for validity and consistency of data values in several fields. For example, fields such as administration route, clinical output, or gender had a predefined set of values inside FAERS structure, so we only had to check its compliance to these options. But data in other fields, such as age, weight, or report date, required to be within certain ranges of valid values and formats. In addition, we built a dictionary for mapping adverse events and indications to Unified Medical Language System (UMLS) codes and gathering a molecular structure dictionary for all user-specified drug names in the main drug or concomitant drugs fields.

After this filtering and curation process, a disproportionality analysis of drug-safety associations was performed using the CLARITY v3.0 platform (Chemotargets SL, Barcelona). To this aim, proportional reporting ratio (PRR) values were obtained by comparing the pool of reports of a given drug with 100 randomly selected pools of the same amount of reports from other drugs. With correlating attributes, such as the distribution of the involved indications and coadministered drugs or the age, weight, and gender of patients, these pools of reports were used as statistical background to mitigate potential confounding effects produced by comparing information for more or less specific niche drugs to reports of drugs that have nothing in common in terms of patient phenotype or concomitant polypharmacy. We employed a customized genetic algorithm to select these backgrounds by matching the distributions of attributes across each set of chosen reports with the distributions coming from the drug related reports. The final PRR value assigned to the drug-safety association is the average PRR value obtained from the 100 independent backgrounds.

For assessing the association of each drug to cardiotoxicity, a list of 29 adverse events was considered, namely, palpitations, central venous catheterization, ventricular fibrillation, sudden cardiac death, myocardial infarction, dilated cardiomyopathy, cardiomegaly, prolonged QT interval, left ventricular hypertrophy, heart murmur, sinus tachycardia, ventricular tachycardia, bradycardia, mitral valve insufficiency, aortic valve insufficiency, tricuspid valve insufficiency, atrial septal defects, ventricular septal defects, torsades de pointes, tachyarrhythmia, cardiac arrhythmia, ejection fraction decreased, cardiotoxicity, right-sided heart failure, cardiogenic shock, coronary heart disease, pericardial effusion, heart diseases, and cardiovascular diseases. For each drug, the major cardiotoxicity event (MCE) was defined as the term, among those 29, with the larger number of reports in FAERS, irrespective of whether the drug was considered a primary suspect, secondary suspect, or concomitant drugs in those reports. A drug was considered FAERS-positive if its MCE had a PRR of 2 or above, a threshold often used in disproportionality analyses (Maignen *et al.*, 2017). In addition, 2 molecules well described as cardiotoxic were included as positives: Doxorubicin, an anthracycline used in chemotherapy that promotes both acute and chronic cardiotoxicity in humans (Raj *et al.*, 2014); and Ibutilide, an antiarrhythmic agent, which can induce or worsen ventricular arrhythmias with fatal consequences (Stambler *et al.*, 1997). On top of the cardiotoxic classification outlined above, we have included 18 molecules withdrawn from the market due to cardiovascular adverse effects. They were collected from the WITHDRAWN resource (Onakpoya *et al.*, 2016; Siramshetty *et al.*, 2016). Finally, compounds have been grouped according to the Anatomical Therapeutic Chemical Classification System code (ATC code, www.whocc.no/atc_ddd_index, Accessed July 30, 2019), ie, organ or system on which they act and their therapeutic, pharmacological and chemical properties.

The 92 molecules tested were then purchased from Prestwick (www.prestwickchemical.com, Accessed July 30, 2019). The Prestwick chemical library is a collection of 1280 small molecules, 95% of which are approved drugs (FDA, EMA, and other agencies), with high chemical and pharmacological diversity and associated bioavailability and safety clinical data. The compounds were obtained as lyophilized powder. The 92 compounds selected from the 1280 compound library were diluted in 100% DMSO (Sigma-Aldrich, St Louis, Missouri, D8418) to a stock concentration of 100 mM for zebrafish testing and 10 mM for hiPSC-CM testing. None of them displayed any solubility issue at both stock concentrations. The list of molecules, with description of their main on-targets, chemical category, FAERS data, year of withdrawal for withdrawn molecules, and concentration (no observed effect concentration; NOEC) used in the zebrafish drug screening are provided in Supplementary Table 1.

#### High-Throughput Screen in hiPSC-CMs

hiPSC-CMs beat spontaneously under culture conditions and the beating pattern can be addressed by Ca^2+^ dyes reporting the intracellular Ca^2+^ concentration that is then measured by the fluorometric imaging plate reader (FLIPR).

##### Preparation of hiPSC-CMs

Pluricyte hiPSC-CMs (NCardia; PCMi-1031-1) were used to perform the *in vitro* screening. hiPSC-CM Pluricytes are suitable for fluorescence-based calcium transient assays using a FLIPR. Culture and maintenance of the Pluricyte hiPSC-CM are described in the online manual of the NCardia website (www.ncardia.com, Accessed July 30, 2019). This standard protocol was changed at 2 steps: hiPSC-CMs were not spun after thawing and the final concentration was 15 000 instead of 10 000 cells/well.

##### FLIPR assay

Twenty microliters of Ca-6 dye solution was added to each well of the assay plate and the assay plate was incubated for 2 h at 37°C, 5% CO_2_ in the dark. Fluorescence was measured (excitation: 470–495/emission: 515–575) to establish the baseline. After baseline measurement, compounds were added and the fluorescence signal was measured 5, 30, and 90 min after compound treatment. Fifteen microliters of compound dilution was transferred from a compound source plate (40 μM, 0.4% DMSO) to the assay plate (already containing 25 μl cells and 20 μl Ca-6 dye) using FLIPR. The final compound concentration was 10 μM and 0.1% DMSO. Between each measurement the plate was returned to the same incubator. Five extracted features were quantified: beating frequency/beats per minute (bpm), amplitude (ampl), area under the curve (auc), peak width (pkw), and peak width at 10% amplitude (ppkw) are measures informing about the depolarization and repolarization kinetics. The entire physical *in vitro* screening was performed by Pivot Park Screening Center (www.ppscreeningcentre.com, Accessed July 30, 2019). Raw data of the experiment were delivered to ZeClinics for analysis. Each compound was assessed as triplicate at 3 time points after incubation (T1: 5 min, T2: 30 min, T3: 90 min). A measurement was taken prior to compound addition (baseline, not shown). As there was little variability in the baseline measurement, it was not necessary to normalize against baseline. An average of the data of each feature for each time point was fed into the statistical model to determine significant effects.

#### Zebrafish Maintenance and Transgenic Lines

Zebrafish (*Danio rerio*) were mated, staged, and raised by standard methods (Westerfield, 2000). The library screen was performed on double transgenic larvae for Tg[*cmlc2*: GFP] (Huang *et al.*, 2003) and Tg[*gata1*: DsRed] (Traver *et al.*, 2003). Tg[*cmlc2*: GFP] expresses green fluorescent protein (GFP) in cardiomyocytes and Tg[*gata1*: DsRed] expresses DsRed fluorescent protein in a subset of erythrocytes.

#### Zebrafish Larvae Preparation for High-Throughput Screen

Determination of NOEC was achieved through an *Acute Toxicity* test performed on AB wildtype larvae from 3 to 96 hpf, as previously described (Cornet *et al.*, 2017) (see Supplementary Table 1 for NOEC data for each compound). For the cardiotoxic drug screening, double transgenic zebrafish larvae for Tg[*cmlc2*: GFP] and Tg[*gata1*: DsRed] were collected and raised in Embryo Medium (E3/60× Stock: 34.4 g NaCl, 1.52 g KCl, 5.8 g CaCl_2_·2H_2_O, 9.8 g MgSO_4_·7H_2_O, add 18 MOhm double distilled water up to 2000 ml; pH: 7.2) at 28.5°C in a 12 h light/12 h dark cycle. From 24 hpf onward, larvae were incubated with N-PhenylThioUrea (PTU) (Sigma-Aldrich, No. P7629) 0.2 mM to reduce pigmentation. Embryo medium was changed every day until 5 dpf, when double positive transgenic larvae were selected. For some compounds the determined NOEC was high (1 mM) and required 1% DMSO concentration. Hence, all compounds were incubated at 1% DMSO regardless of their NOEC to standardize conditions among all tested molecules. Larvae were immersed in E3 with PTU 0.2 mM and Tricaine methanesulfonate (Sigma-Aldrich, No. E10521) at 168 mg/ml working concentration and distributed in 96-well plates in 100 µl per well. Then, 100 µl of compound solution was added at double NOEC concentration to reach the working concentration per compound: NOEC, DMSO 1%, and the final volume of 200 µl per larva per well. Negative control larvae were incubated in 200 µl 1% DMSO. Embryos were allowed to incubate in the compound solution for 4 h before. The developmental stage selected for analysis (5 dpf) was chosen because heart electrophysiological maturation and heart rhythm are stabilized from 96 hpf (Tessadori *et al.*, 2012). One hundred and twenty hours post fertilization constitutes the latest stage to perform analysis after heart maturation and with conformation to ethical guidelines. Finally, the drug incubation time of 4 h was selected according to a previously established experimental set up (Cornet *et al.*, 2017). Twelve embryos (1 embryo/well) were imaged for each compound, and the screening of the whole library was distributed over 15 experimental days. On each experimental day, 8 molecules were imaged: 7 drugs and the negative control 1% DMSO, to allow intraexperimental comparison. The screening resulted in approximately 1500 videos, which were organized and analyzed by the ZeCardio software.

#### ZeCardio Screening Platform

##### Hardware

The hardware conforming the screening platform allows zebrafish larvae sampling from multi-well plates to allow their position and automatic orientation in a glass capillary under a microscope to perform high-throughput imaging acquisition. It integrates 2 commercially available systems. First, an integrated VAST system (Union Biometrica) (Pardo-Martin *et al.*, 2010), which consists of 2 complementary devices: (1) LP sampler, an automated fluid system capable of aspirating small to medium sized particles from multi-well plates and delivering them intact to the VAST BioImager, and (2) VAST BioImager, an automated fluid system, which positions zebrafish larva in a glass capillary. Once introduced in the glass capillary, the larva is recognized by an integrated imaging system and rotated to the desired angle for external imaging. Second, a Leica DM6B upright widefield microscope with a motorized Z-stage and Leica DFC9000 GTC (4.2 megapixel) CMOS camera particularly suited for high-speed fluorescent imaging applications achieves the fluorescent time-lapse imaging. The 2 systems, VAST and Leica microscope, communicate to coordinate the tasks of sampling, positioning, and imaging. To control the microscope, the LASX software (Leica) was used. To focus fluorescent structures in z-direction, the *Autofocus* function in LASX was used. Time-lapse movies were acquired with a 10× water-dipping lens (NA: 0.4) at binning 2× (effective pixel size 1.23 µm). Videos of the zebrafish heart and vasculature were acquired at a frame rate of 75.9 frames per second (fps) and 70.5 fps, respectively. Each time-lapse movie had a duration of 40 s. Video data were saved in .lif format. The integrated hardware can be employed to perform fully automated imaging of the entire 96-well plate (Supplementary Figure 1).

##### ZeCardio software

The time-lapse movies were converted from .lif format to .zecardio format and imported in the ZeCardio software. The ZeCardio software, developed by ZeClinics, provides a graphical user interface (GUI) that facilitates organization of large amounts of data and the quantification of cardiovascular features from heart and vasculature videos.

The analysis in ZeCardio is semiautomatic and depends on the user to indicate the location of a heart or of a blood vessel. For cardiac feature quantification the user draws a line along the heart axis, from ventricle to atrium to initiate the calculation. Two additional lines perpendicular to the heart axis, at the level of ventricle and atrium are automatically displayed. All lines can be subjected to modification of their angles and lengths. From the line selections, 2 types of output are generated: (1) A kymograph for each of the perpendicular lines (ie, for each heart chamber) that on one hand allows the visual inspection and easy identification/validation of phenotypes, and on the other hand is used for individual beat detection. (2) A quantification of features computed from the videos at according to the drawn structures. These numerical outputs are directly computed displayed in the ZeCardio GUI. Several features can be quantified from heart videos: The Heart beat frequency is detected for each heart chamber and frequencies are presented in the GUI as an average. A boxplot of the distribution of lengths of individual beats indicates beat length stability over time. The percentage of arrhythmia is shown in percent. Chamber specific cardiac arrest is measured in seconds as the longest period without beating. Additionally, Non-beating chambers can be flagged manually by the user. For calculation of the QTc interval (linearly corrected QT interval), the Framingham formula (QTc = QT + 0.154 (1 − RR)) was adjusted for zebrafish larvae as QTc = QT + 0.154 (2.66 − RR). RR = 6.6 ms/bpm is applied. Ejection fraction, the fraction of blood ejected upon ventricle contraction, is estimated from the maximal dilatation (diastolic diameter) versus the maximal contraction (systolic diameter) and is expressed in %. The physical length of heart chambers is displayed in µm. Very specific phenotypes such as AV Coupling defects, in particular Bigeminy and Trigeminy can be flagged by the user if seen. As for heart videos the quantification of vasculature features relies on the analysis of the kymograph. The velocities of blood cells are analyzed and ZeCardio reports the average and median velocities. The 10th and 90th percentile of the distribution correspond to systolic and diastolic velocities. As for the heart the user can flag “no flow” for arteries and veins. All of the computed and flagged values can be exported in .csv format for further analysis (Supplementary Figure 2).

**Figure 1.**
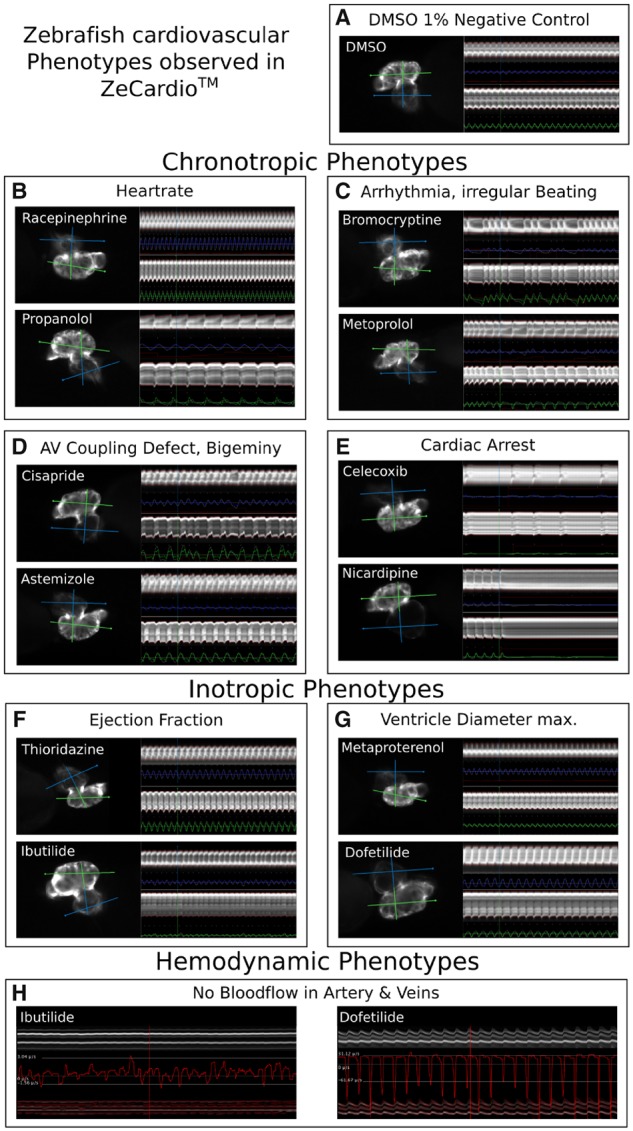
Cardiovascular phenotypes in zebrafish as visualized in ZeCardio. A selection of representative examples of the cardiovascular phenotypes observed in zebrafish larvae is shown. The individual panels show screen-shots of the display of compounds in the ZeCardio software. For comparison of the cardiac phenotypes (B–G) a heart of negative control larva (DMSO 1%) is shown (A). Chronotropic phenotypes include increase (tachycardia, Racepinephrine, B, top) and decrease (bradycardia, Propranolol, B, bottom) of the heart rate, irregular beating of the heart (arrhythmia, Bromocriptine, Metoprolol, C), AV Coupling defects (Cisapride, D, top) and the special case of Bigeminy (Astemizole, D, bottom) and events of cardiac arrest (Celecoxib and Nicardipine, E). Inotropic phenotypes include changes in ejection fraction (increased ejection fraction: Thioridazine, F, top, decreased ejection fraction, Ibutilide, F, bottom) and changes in the size of the ventricle (increase of the maximum ventricle diameter, Metaproterenol, G, top, and decrease of the maximum ventricle diameter, Dofetilide, G, bottom). In each panel of cardiac phenotypes (A–G) the heart is shown on left and the compound is indicated in the upper left corner. The heart selection in ZeCardio is shown: The quasi vertical line is the selection drawn by the user in ZeCardio. The green perpendicular line is the ventricle line and the blue is the atrium line. The kymographs for ventricle and atrium are shown on the right hand side of each heart panel. Note that for simplicity the kymograph of the atrium is always shown on the top and the kymograph of the ventricle always on the bottom, independent of the heart orientation. Below the individual kymographs a colored line indicates the inverse length of the chamber (ventricle in green, atrium in blue) such that peaks of that curve indicate contractions of the chambers. The hemodynamic phenotypes considered here include absence of blood flow in arteries and veins (H). The 2 panels (Ibutilide, left and Dofetilide, right) show the blood flow profile of the same embryos shown in (F, bottom and G, bottom). Only the kymograph of the vein selection is shown. Below the kymograph, its segmentation is shown (red outlines of the kymograph, bottom of the panel) and the velocities of blood cells are displayed as a red graph. Note that the velocities in Dofetilide reflect the rhythmic nature of the heart beat but that there is no net directional flow, ie, blood cells merely move back and forth in the vessel.

For generating the cardiotoxic profile of compounds in zebrafish larvae the numerical features assessed were: ventricular beat rate (ventricular BPM), corrected QT interval (QTc), longest time without beating (cardiac arrest), percentage of arrhythmic beats (arrhythmic beats), estimated ejection fraction (ejection fraction), and maximal diameter of ventricle—ie, the diameter of the ventricle in diastole—(max. diameter). As such, we have selected examples of altered chronotropic, inotropic, and hemodynamic features. Figure 1 and Supplementary Movie 1 show representative examples of compounds displaying these features. Figure 1A shows an example of a normally beating heart (1% DMSO, negative control), which displays a clear difference to chronotropic phenotypes such as heart rate increase (tachycardia, Racepinephrine) and decrease (bradycardia, Propranolol) (Figure 1B). Examples of arrhythmia (ie, irregular beating), as a chronotropic effect, is shown with Bromocriptine and Metoprolol (Figure 1C). AV Coupling defects and Bigeminy are shown with Cisapride. Inotropic phenotypes, such as increased and decreased ejection fraction, are shown for Thioridazine and Ibutilide, respectively (Figure 1E). An increase and decrease of the maximum ventricle diameter are shown for Dofetilide and Metaproterenol, respectively (Figure 1F). Although we did observe heart beating in Ibutilide and Dofetilide, those embryos showed a dramatically disrupted blood flow in both artery and vein (vein shown for Ibutilide and Dofetilide, artery not shown, Figure 1G). Furthermore, the ZeCardio flags considered for generating the cardiotoxic profile of compounds comprise AV Coupling Defects, Bigeminy (a special case of AV Coupling defect with rhythmic ectopic beats, one after each sinus beat), complete absence of heart beating (no beating) and absence of arterial or venous blood flow (artery no flow, vein no flow). We classified those features as positive for a compound when incidence (ie, the percentage of larvae labeled with that flag) was above 30%.

#### LME Statistical Model

To evaluate which compounds can significantly alter the different parameter responses we applied linear mixed effects models (LMMs). These type of models have been shown to deal with zebrafish larvae experiments where response parameters are arranged in multi-well plate layouts, and thus provide more efficient alternatives to classical statistical approaches (Liu *et al.*, 2017). The LMMs include random effects accounting for the different sources of variation from the experimental design.

Assumed that $y_{\mathrm{irpj}}$ is the response observation of the *j*th embryo from *i*th tested compound, the model is specified as follows:

$$y_{\mathrm{irpj}}=\mu +\beta_{r}+\gamma_{p}+\alpha_{i}+e_{\mathrm{irpj}},$$

where μ is the general mean, $\beta_{r}$ is the row location fixed effect (embryos located at same row across plates have same $\beta_{r}$ effect size), $\gamma_{p}$ is a plate-specific random effect and accounts for between plate variation (embryos located in the same well-plate/imaged on the same experimental day/belonging to the same spawn are correlated), and $\alpha_{i}$ is the random effect of compound *i* and accounts for between compound variation (embryos exposed to the same compound are correlated). $\left\{\alpha_{i}\right\}$ and $\left\{\gamma_{p}\right\}$ are independent $\;N\left(0,\sigma_{\alpha }\right)$ and $N\left(0,\sigma_{\gamma }\right)$, respectively. $e_{\mathrm{irpj}}$ is the random error with independent $N\left(0,\sigma_{e}\right)$. Under the assumptions of the model, compounds that significantly alter each cardiovascular parameter are evaluated by comparing specific effect of each component ($\alpha_{i}$) to the DMSO effect ($\alpha_{\mathrm{DMSO}}$). False discovery rate was used to control type I error rate accounting for the multiple compound based comparisons, and resulting adjusted *p*-values lower than .05 are considered as statistically significant. For zebrafish ventricular beat rate we choose an adjusted *p*-value cutoff of .0013. This value resulted from the calibration detection rates over the range of adjusted *p*-values between 0 and .05. As in zebrafish the physiologically normal range of heart rate variability is much greater than in humans we chose this more stringent cutoff to better discriminate cardiotoxicity from cardio-modulation (see Results section).

For visualization purposes we construct a *z*-score as follows:

$$z_{i\;\mathrm{vs}\;\mathrm{DMSO}}=\frac{\alpha_{i}-\alpha_{\mathrm{DMSO}}}{\sqrt{{\sigma_{i}}^{2}+{\sigma_{\mathrm{DMSO}}}^{2}}}.$$

**Figure 2.**
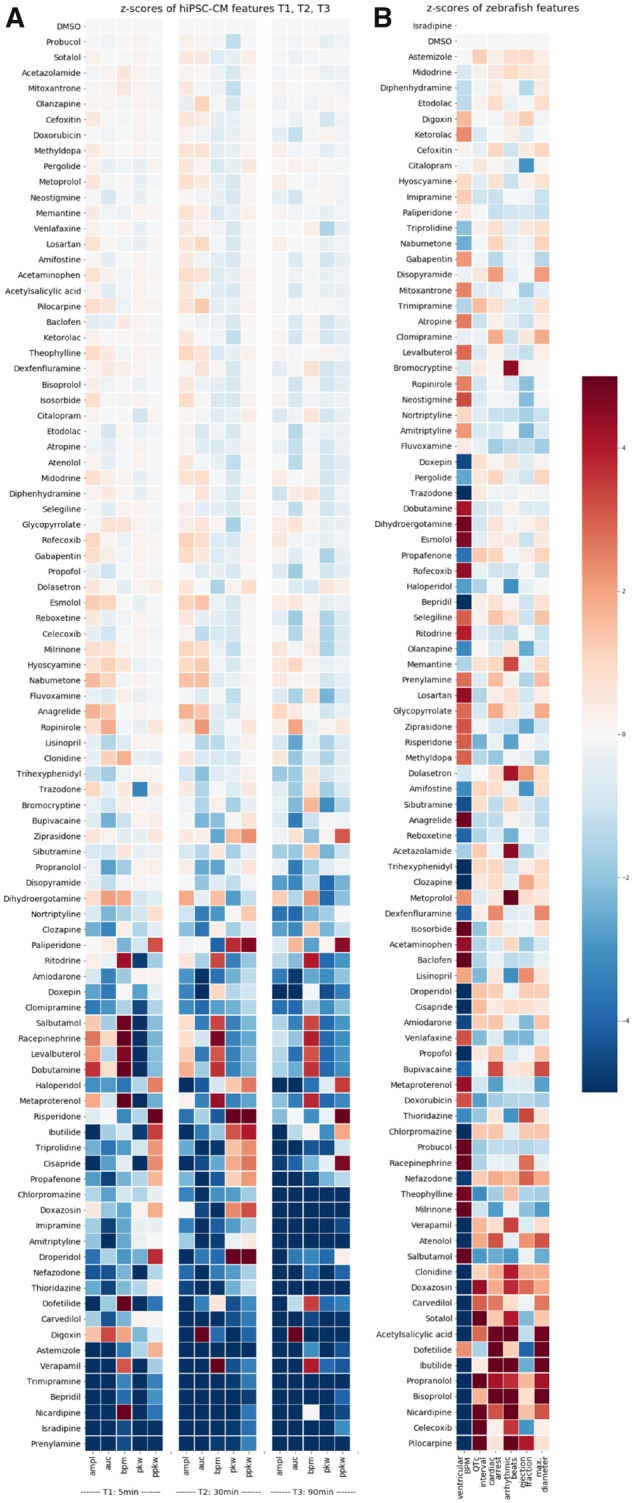
Heatmap of the *z*-scores of human induced pluripotent stem cell cardiomyocyte (hiPSC-CM) and zebrafish features. A, The *z*-scores of features measured by fluorometric imaging plate reader (FLIPR) in hiPSC-CMs at 5 min (T1), 30 min (T2), and 90 min (T3) after compound addition are shown for each compound of the library: amplitude (ampl), area under curve (auc), beats per minute (bpm), peak width (pkw), and peak width at 10% amplitude (ppkw). The *z*-scores were computed from the effects determined by the statistical model for each compound and each time point. B, The *z*-scores of numerical features in zebrafish larvae 4 h after compound addition quantified by ZeCardio and analyzed by the statistical model are shown for each compound of the library. Six features are shown: ventricular beats per minute (ventricular BPM), QTc interval (QTc), longest beat (cardiac arrest), percentage of arrhythmic beats (arrhythmic beats), estimated ejection fraction (ejection fraction), and the maximum ventricular diameter (max. diameter). All cells are color-coded according to their *z*-score (color bar on the right hand side) and compounds in (A) or (B) are ordered according to the sum of absolute *z*-scores for each compound from bottom to top.

This *z*-score is assumed then to follow a standard normal distribution (*z*-score ∼$\;N\left(0,\;1\right)$), and therefore extreme values of this score are considered as “toxic” (Figure 2). An exception is the zebrafish features arrhythmic beats and cardiac arrest. Here only increases and not decreases can be considered. Therefor we labeled compounds as positive for these features only if they had a positive *z*-score. R was used for the statistical analysis. Data preprocessing and visualization were done with Python.

## RESULTS

The cardiotoxic profile of all compounds in the hiPSC-CM and zebrafish larva model is depicted as a heatmap of *z*-scores (Figure 2). A table of the *z*-scores is provided in Supplementary Table 2. For each model system the compounds are ordered according to the sum of absolute *z*-scores of the different features. In hiPSC-CMs, decreases in respect to the negative control DMSO were more commonly observed than increases (compare amount of blue and red cells in Figure 2A). The strength of parameter responses at the individual time points (T1, 5 min; T2, 30 min; T3, 90 min post incubation) appears to be correlated for the majority of compounds. Additionally, parameter responses across the 3 different time points are rather correlated as well. However, some compounds show a stronger effect at the early time point (T1, 5 min; eg, Nicardipine, Dofetilide, Digoxin), others at midrange (T2, 30 min, eg, Verapamil, Ibutilide, Bepridil), yet others show a late effect (T3, 90 min, eg, Bromocriptine, Thioridazine). In zebrafish larvae no clear correlation of the parameter response strength of different numerical features could be observed (Figure 2B). For the features cardiac arrest and arrhythmic beats, only positive effects can be considered given the nature of those features: a decrease in arrhythmic beats or cardiac arrest can hardly be considered pathological. Interestingly, the feature ventricular BPM showed a significant effect in most of the compounds. Although the visualization of parameter responses illustrates well the cardiotoxic profile of compounds in the 2 models, a subsequent discrimination into positive and negative compounds had to be done for cross-comparison with human data. For hiPSC-CMs any compound displaying a significant effect for any of the features at any of the time points was classified as positively detected. This yielded 47 detected and 45 nondetected compounds (Table 1). For the zebrafish model we classified as positive all compounds with a significant effect in any of the numerical features or with an incidence of 30% and above for any of the binary features (ie, ZeCardio flags). As mentioned above, the ventricular BPM feature showed a statistically significant effect for the vast majority of compounds. Given the chemical diversity of the library, it is possible that the causes are general chemico-physical features rather than drug on/off-target activities. Given that the electrical conduction system of the heart relies on ion channels (Grant, 2009), addition of drugs might promote pH changes that might result in ion channel disturbances (Peters *et al.*, 2018). In addition, the zebrafish is exposed to large temperature and saline variations in its natural habitat; it is likely that the significant changes we observe in ventricular beat rate are well within the normal physiological range. To better calibrate the zebrafish detection system we applied a more stringent statistical threshold for this feature (adjusted *p*-value ≤ .0013) in order to achieve biologically meaningful detection of cardiotoxicity. The classification of compounds after calibration of the detection system yielded 60 detected and 32 nondetected compounds (Table 1).

**Table 1.** Detection Rates of Zebrafish and hiPSC-CM Models for the Compound Library and Subgroups

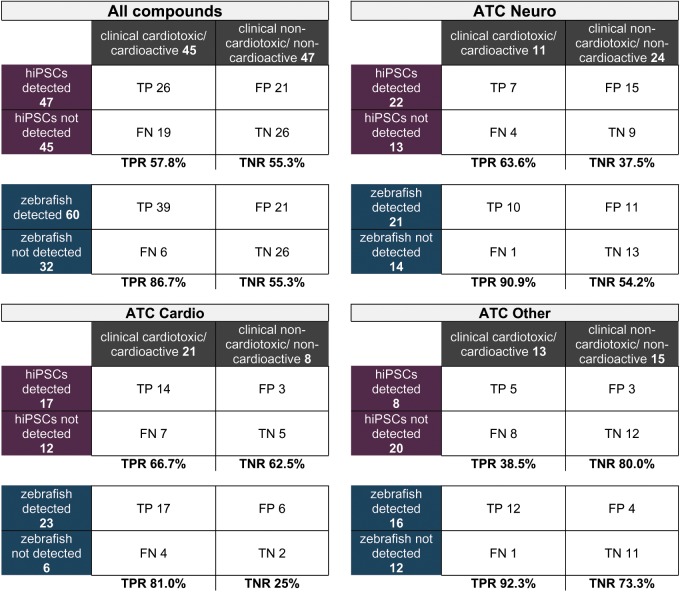

2 × 2 contingency tables are shown for the entire compound library and for each of subgroups (ATC-Cardio, ATC-Neuro, and ATC-Other) indicating the number of true positives (TP), true negatives (TN), false positives (FP), and false negatives (FN) and the resulting true positive rate (TPR) and true negative rate (TNR) displayed in bold lettering for hiPCS/CMs (purple) and zebrafish larvae (blue). The number of detected and nondetected molecules is indicated for each model and for the clinical data (grey). TPR = TP/TP + FN; TNR = TN/TN + FP.

Of the 45 positive compounds in hiPSC-CMs, only 10 (2 true positives [TPs] and 8 false positives [FPs]) showed a differential effect in detection at individual time points. The remaining 35 molecules were positive for all 3 time points assessed. The 2 TPs detected only at T3 (90 min past incubation) are the withdrawn molecules Sibutramine and Bromocriptine, leading us to conclude that imaging late after compound addition is beneficial in order to reach better sensitivity. Of the 60 compounds detected in zebrafish, 11 molecules (4 TPs and 7 FPs) were detected due to features other than changes in ventricular BPM. We conclude that the assessment of those features is crucial for predicting human cardiotoxicity as the 4 TP molecules detected for effects in features other than BPM are all withdrawn drugs (Dofetilide, Bromocriptine, Astemizole, and Dolasetron).

**Figure 3.**
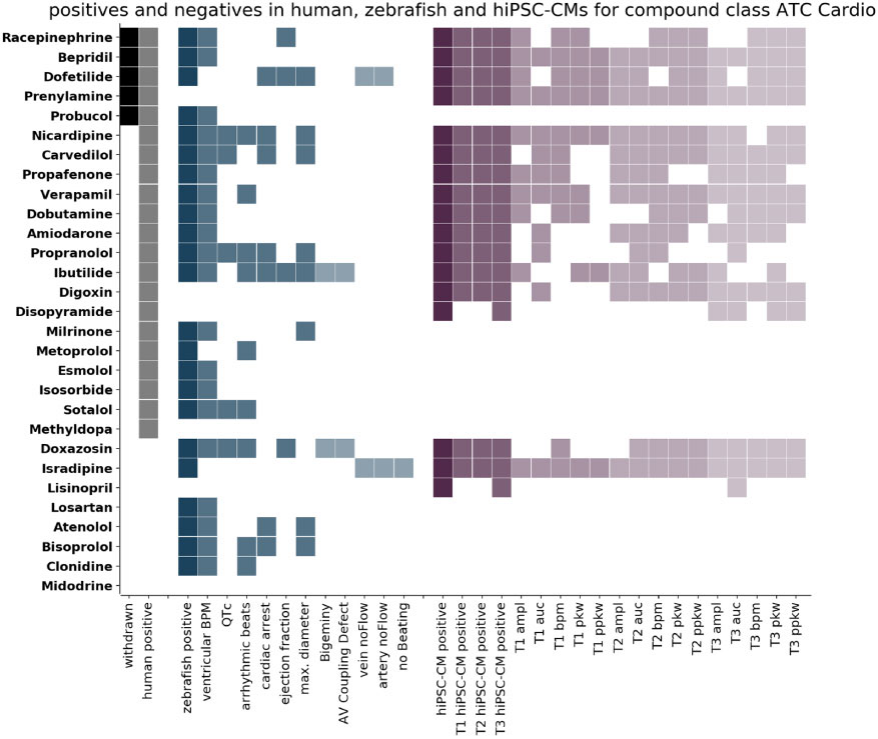
Comparison of positives in humans, zebrafish larvae, and human induced pluripotent stem cell cardiomyocytes (hiPSC-CMs) for compounds of the class ATC-Cardio. The compounds of class ATC-Cardio are listed on the left. Each row corresponds to 1 compound and the colored cells indicate detection of that compound. Columns correspond to the criteria stated on the x-axis. Compounds are classified as positive in humans (human positive, grey) if they are either withdrawn (withdrawn, black) or have been classified positive on the basis of FAERS data. Compounds are classified as positive in zebrafish (zebrafish positive, dark blue) if significant effects have been detected for any of the numerical features (blue) or if the incidence of Boolean features was above threshold (light blue). Compounds are classified as positive in hiPSC-CMs (hiPSC-CM positive, dark purple) if significant effects have been detected for any of the features at any of the time points (T1 5 min, T2 30 min, T3 90 min after compound addition). The columns T1 hiPSC-CM positive, T2 hiPSC-CM positive, and T3 hiPSC-CM positive (purple) indicate whether a compound was detected at this time point and the detection of the single features for the 3 time points are displayed (light shades of purple). The compounds are ordered according to their response in humans from top to bottom.

**Figure 4.**
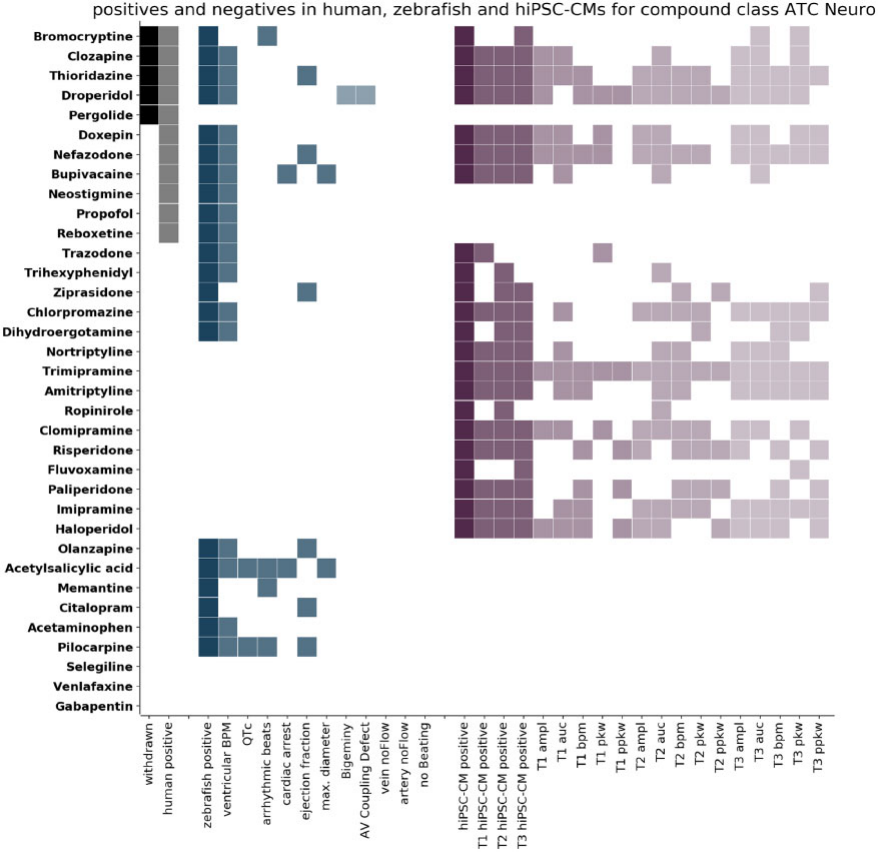
Comparison of positives in humans, zebrafish larvae, and human induced pluripotent stem cell cardiomyocytes (hiPSC-CMs) for compounds of the class ATC-Neuro. The compounds of class ATC-Neuro are listed on the left. Each row corresponds to 1 compound and the colored cells indicate detection of that compound. Columns correspond to the criteria stated on the x-axis. Compounds are classified as positive in humans (human positive, grey) if they are either withdrawn (withdrawn, black) or have been classified positive on the basis of FAERS data. Compounds are classified as positive in zebrafish (zebrafish positive, dark blue) if significant effects have been detected for any of the numerical features (blue), or if the incidence of Boolean features was above threshold (light blue). Compounds are classified as positive in hiPSC-CMs (hiPSC-CM positive, dark purple) if significant effects have been detected for any of the features at any of the time points (T1 5 min, T2 30 min, T3 90 min after compound addition). The columns T1 hiPSC-CM positive, T2 hiPSC-CM positive, and T3 hiPSC-CM positive (purple) indicate whether a compound was detected at this time point and the detection of the single features for the 3 time points are displayed (light shades of purple). The compounds are ordered according to their response in humans from top to bottom.

**Figure 5.**
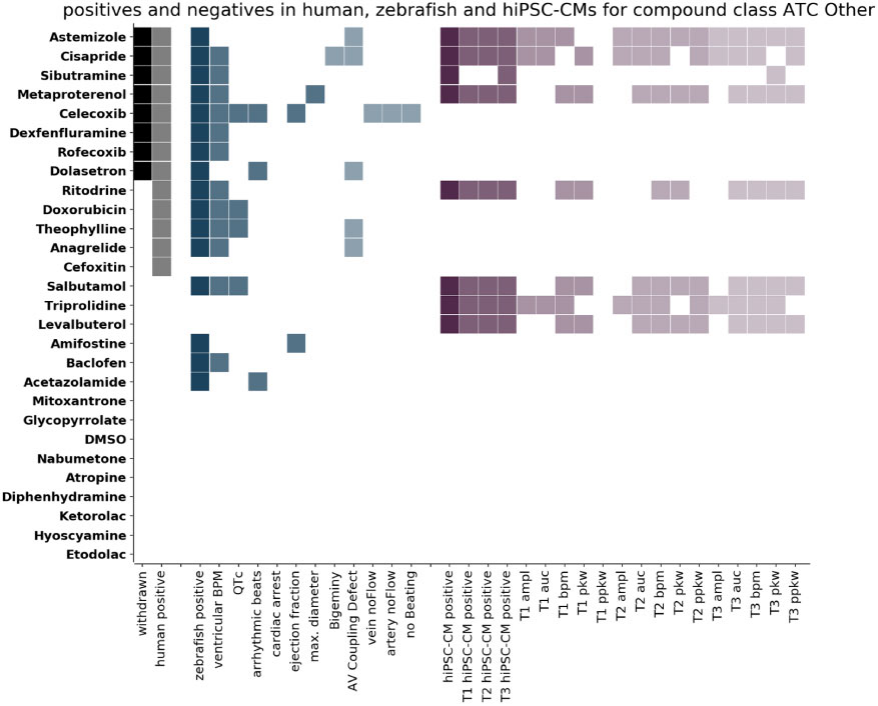
Comparison of positives in humans, zebrafish larvae, and human induced pluripotent stem cell cardiomyocytes (hiPSC-CMs) for compounds of the class ATC-Other. The compounds of class ATC-Other (belonging to neither ATC-Cardio nor ATC-Neuro) are listed on the left. Each row corresponds to 1 compound and the colored cells indicate detection of that compound. Columns correspond to the criteria stated on the x-axis. Compounds are classified as positive in humans (human positive, grey) if they are either withdrawn (withdrawn, black) or have been classified positive on the basis of FAERS data. Compounds are classified as positive in zebrafish (zebrafish positive, dark blue) if significant effects have been detected for any of the numerical features (blue) or if the incidence of Boolean features was above threshold (light blue). Compounds are classified as positive in hiPSC-CMs (hiPSC-CM positive, dark purple) if significant effects have been detected for any of the features at any of the time points (T1 5 min, T2 30 min, T3 90 min after compound addition). The columns T1 hiPSC-CM positive, T2 hiPSC-CM positive, and T3 hiPSC-CM positive (purple) indicate whether a compound was detected at this time point and the detection of the single features for the 3 time points are displayed (light shades of purple). The compounds are ordered according to their response in humans from top to bottom.

Our main aim was to address if the 2 experimental models respond differently according to a compounds target tissue or its MoA. For this, we grouped the compounds into 3 categories: ATC-Cardio (cardioactive molecules, Figure 3), ATC-Neuro (neuroactive molecules, Figure 4), and ATC-Other (all remaining ATC classes, Figure 5) and displayed the clinical information (withdrawn molecules [black cells, first column], human cardiotoxic liabilities [grey cells, second column]) against the general classification of cardiotoxicity and the specific features detected in both models (zebrafish: blue shades, hiPSC-CMs: purple shades). Compounds were ordered top to bottom according to the withdrawn and positive cardiotoxic clinical classification. This visualization allowed the inspection of detected responses in zebrafish larvae and hiPSC-CMs and the cross-comparison of clinical and experimental information.

### 

#### Cardioactives, ATC-Cardio

The ATC-Cardio subgroup (Figure 3) comprises 29 molecules of which 21 display cardiotoxic concerns (Table 1); 5 of those were withdrawn from the market (Bepridil, Dofetilide, Prenylamine, Probucol, and Racepinephrine). In the ATC-Cardio categories are adrenergic antagonists and agonists, Ion channel inhibitors, and other cardio-modulators. Cardiotoxicity promoted by compounds of the ATC-Cardio class is likely due to on-target-based toxicity and both models should respond similarly when exposed to drugs targeting cardiac cells. In hiPSC-CMs we detected 17, in zebrafish 23 molecules as positive. The TPR (calculated as TPR = TP/TP + False Negatives (FN)) for the hiPSC-CM model and the zebrafish model are 66% and 80%, respectively. The TNR (calculated as TNR = True Negatives (TN)/TN + False Positives (FP)) for hiPSC-CMs and zebrafish are 62% and 25%, respectively (Table 1). Of the 5 withdrawn molecules, the zebrafish model allowed the detection of all of them except Prenylamine, a myosin-light chain kinase 2 inhibitor and calmodulin modulator which was used as antiarrhythmic. The hiPSC-CM model failed to detect Probucol, an ATP-binding cassette subfamily A member 1 inhibitor. Both models show 80% sensitivity in the detection of withdrawn molecules.

#### Neuroactives, ATC-Neuro

The ATC-Neuro subgroup (Figure 4) includes 35 compounds, among them tricyclic antidepressants (TCA), selective serotonin reuptake inhibitors (SSRI), Serotonin-Norepinephrine reuptake inhibitors (SNRI), dopamine and serotonin (5-HT) receptor antagonists and other neuronal receptor modulators. The impact of the autonomous nervous system in the vertebrate cardiovascular function is well known (Shen and Zipes, 2014; Gordan *et al.*, 2015). The sympathetic and parasympathetic system are conserved in zebrafish (Mann *et al.*, 2010), whereas hiPSC-CM cultures are isolated from autonomous nervous system cues. Thus, it is possible that the experimental models show a differential performance in predicting cardiotoxicity for ATC-Neuro compounds. Of the 35 molecules, 11 have been labeled positive in human. Five of these human positives were withdrawn from the market (Bromocriptine, Clozapine, Droperidol, Pergolide, and Thioridazine). In zebrafish we detected 21, in hiPSC-CMs 22 compounds as positives. This led to the following rates: TPR 90% and TNR 54% for zebrafish and TPR 63% and TNR 37% for hiPSC-CM (Table 1). Of the 5 withdrawn molecules, both zebrafish and hiPSC-CMs failed to detect Pergolide, a Dopamine receptor Agonist (80% sensitivity for both models).

#### Other Therapeutic Classes, ATC-Other

The subgroup ATC-Other (Figure 5) consists of 28 compounds with different ATC classes, such as ATC-A (Alimentary tract and Metabolism), ATC-D (Dermatologicals), ATC-M (Musculo-skeletal system), ATC-R (Respiratory), and ATC-L (anti Neoplastic). Thirteen of those compounds were positive in humans. Of those molecules, 8 were withdrawn from the market due to cardiotoxicity concerns (Astemizole, Celecoxib, Cisapride, Dexfenfluramine, Dolasetron, Metaproterenol, Rofecoxib, and Sibutramine). We classified as positive 16 molecules in zebrafish and 8 in hiPSC-CMs. This led to a TPR of 92% and a TNR of 73% for zebrafish, and a TPR of 38% and a TNR of 37% for hiPSC-CMs. Of the withdrawn molecules, the zebrafish model could detect all (100% sensitivity), whereas hiPSC-CMs failed to detect Celecoxib and Rofecoxib—both Cox inhibitors; Dexfenfluramine—a serotonin–norepinephrine releasing agent (SNRA); and Dolasetron—a 5-HTR & serotonin receptor antagonist (50% sensitivity).

#### Analysis of the Entire Library

After assessing the predictive potential for compound groups according to their therapeutic application, it was important to compare the general predictive performance of both models regardless of the target tissue or potential mechanism of toxicity. In the entire library, 45 molecules are classified positives in humans and 47 as negatives. In zebrafish we detected 60 and in hiPSC-CMs 47 molecules as positive (Table 1). The detection rates were as follows: TPR of 86% and TNR of 55% for zebrafish, and TPR of 57% and TNR of 55% for hiPSC-CM. Regarding the 18 withdrawn molecules, 16 were detected in zebrafish (Sensitivity of 89%) and 12 in hiPSC-CMs (Sensitivity of 67%).

## DISCUSSION

We have developed ZeCardio, an integrated screening platform that provides access to complex chronotropic, inotropic, and hemodynamic phenotypes in zebrafish. A powerful use of this platform is the prediction of drug-induced human cardiovascular toxicity. To validate the platform, and this potential application, we have performed a cross-comparison of the predictive performance to unveil human cardiotoxic liabilities between the *in vivo* zebrafish model and a traditional *in vitro* hiPSC-CM model.

In general, the zebrafish model yielded a much higher sensitivity than the hiPSC-CM model (Table 1). This approximately 30% difference in TPR is explained by the identification of 13 zebrafish TPs not detected with hiPSC-CMs. Metabolic reasons might explain this divergence; hiPSC-CM cultures are isolated from the metabolism, whereas zebrafish has a mature metabolism at the stage coinciding with the screening (Goldstone *et al.*, 2010). Many drugs target metabolic enzymes which may differentially regulate ADME of drugs enhancing cardiotoxicity (Solanki *et al.*, 2018). This might explain why only zebrafish recognize Rofecoxib, whose metabolite glucuronidated 5-hydroxyrofecoxib promotes the cardiovascular clinical adverse effects—heart attack and stroke—that led to its market withdrawal (Zhang *et al.*, 2012). Interestingly, TP molecules for both models, such as Astemizole and Sibutramine, are also metabolized, but they promote cardiovascular clinical adverse effects through both the parent drug and metabolites (Zhang *et al.*, 2012). On the other hand, both models displayed a medium specificity, which resulted from an overrepresentation of FP compounds. Two factors might explain this high FP rate in zebrafish. First, drug incubation time may be too long for some compounds, leading to overaccumulation in zebrafish tissues. In the same line, NOEC might be too high, when compared to human blood concentrations promoting clinical cardiovascular phenotypes. Certainly, high exposures in mammalian models, when compared to human biodisponibility data, lead to a higher FP rate (Monticello *et al.*, 2017). Second, our criterion for selecting human cardioactive compounds has been very stringent. Indeed, the enrichment of safety terms above noise might be subjected to some bias. For example, a recent report monitoring the evolution over time of adverse event signals uncovered that side effects were more often reported for drugs in the news, whereas drugs with low or discontinued clinical use displayed lower than expected safety reporting (Maciejewski *et al.*, 2017). This might have resulted in the exclusion of a number of *bona fide* cardiotoxic compounds and, hence, their classification as FPs by both models.

In hiPSC-CMs, we have screened at 3 different time points after compound addition. Although hiPSC-CM responses are mostly similar among individual time points, our results show that 2 withdrawn drugs (Sibutramine and Bromocriptine) were only detected as cardiotoxic through the late assessment. This suggests that longer incubation times allow better predictivity. In zebrafish, the assessment of features other than ventricular BPM enabled the detection of 4 withdrawn molecules (Dofetilide, Bromocriptine, Astemizole, and Dolasetron). This highlights the importance of taking these features in consideration when it comes to unveiling cardiotoxic liabilities.

To uncover the predictive performances of the experimental models for distinct compound classes we divided the entire library into the 3 subgroups ATC-Cardio, ATC-Neuro, and ATC-Other. For cardiovascular drugs (ATC-Cardio), zebrafish outperforms hiPSC-CMs in sensibility, but has lower specificity due to the higher number of FPs. There are 14 adrenergic modulators among the compounds in the ATC-Cardio category. Zebrafish detects 12 of them (4 as FPs), whereas hiPSC-CMs detect 6 (6 TPs). Adrenergic signaling is crucial in cardiac function (Madamanchi, 2007), with adrenergic β-receptors (especially β1) primarily expressed in cardiac tissue whereas α-receptors (especially α1 and α2) primarily expressed in smooth muscle cells of the vasculature. Our results indicate that zebrafish is more sensitive to detect adrenergic modulation than hiPSC-CMs. On one hand, that might be explained by the low differentiation state of hiPSC-CMs, and the fact that adrenergic signaling increases upon hiPSC-CM maturation (Brito-Martins *et al.*, 2008). On the other hand, alpha adrenergic signaling can act either directly on myocardial alpha-adrenoreceptors to mediate positive inotropy or indirectly via hemodynamic effects (Long and Kirby, 2008).

Another extensive group of tested molecules are ion channel inhibitors—9 molecules in the ATC-Cardio subset and 12 in the entire library. The majority (8) is Calcium channel inhibitors, the remainder inhibits Potassium and Sodium channels. Ten of the 12 ion channel inhibitors have been labeled cardiotoxic in humans and, remarkably, the sensitivity in detecting cardiotoxic liabilities of those drugs is the same for both models (9 TPs). A strong argument for the use of hiPSC-CMs in preclinical screens is the genetic conservation of components of the cardiac conduction system. Our results indicate that the genetic conservation of zebrafish is sufficient to assess the safety of molecules targeting ion channels. Finally, of the 5 withdrawn drugs in the ATC-Cardio category hiPSC-CMs cannot detect Probucol. This cholesterol lowering agent and potent antioxidant is known to interfere with hERG activity in human patients and rat cardiomyocytes (Guo *et al.*, 2007; Shi *et al.*, 2015). The fact that Probucol does not show arrhythmic phenotypes in hiPSC-CMs might be due to the low concentration tested, lack of cellular differentiation regarding the hERG system or other aspects in the experimental set up. Zebrafish larvae failed to detect Prenylamine, a vasodilator, whose primary target is MLCK2. The role of MLCK2 during embryonic development (Matson *et al.*, 2005) has likely been responsible for the strong developmental defects promoted by higher concentration in the acute toxicity test. That prompted to use a working concentration that might have been too low for promoting cardiotoxicity. Indeed, it would be interesting to test increasing concentrations of this molecule to confirm its cardiotoxic impact in zebrafish.

When considering neuroactive compounds, zebrafish superseded hiPSC-CMs in sensitivity and specificity. As before, the main caveat for zebrafish is that specificity was burdened by the number of FPs. The ATC-Neuro group contains several neurotransmitter transport inhibitors. Cardiotoxicity from this group of compounds might stem from off-target effects on cardiac ion channels and from disturbances of the autonomous nervous system, which can lead to life threatening arrhythmias and heart failure (Gordan *et al.*, 2015). Our results underline the importance of the interplay of heart and Central Nervous System (CNS) to provide a more accurate prediction of cardiotoxicity. Of the 5 withdrawn drugs in the ATC-Neuro category neither model detected Pergolide, a dopaminergic receptor antagonist, withdrawn from the market for promoting valvulopathies. It is plausible that hiPSC-CMs would not detect drugs affecting distinct human anatomical features. For the zebrafish model, we did not assess valvular phenotypes specifically, but the presence of these structures in the zebrafish heart should certainly allow to address these questions in the future.

Finally, for the ATC-Other subclass, the differences between the zebrafish and hiPSC-CM models are even more pronounced. Zebrafish outperformed hiPSC-CM in sensitivity and had a similarly high specificity. Additionally, zebrafish detected all withdrawn molecules (8/8), whereas hiPSC-CMs did not even detect half of them. Two of those, Celecoxib and Rofecoxib, are nonsteroidal anti-inflammatory drugs (NSAIDs) inhibiting COX-2. Importantly, nonaspirin NSAIDs have been given a cardiovascular black-box warning by the FDA from 2015 (Bello and Holt, 2014). The discrepancy in sensitivity of the 2 models might be due to the fact that COX-2 expression in human cardiomyocytes is normally low, unless induced by stress or inflammatory signals (Turini and DuBois, 2002), whereas zebrafish larvae show constitutive expression of COX-2 in heart and brain (Grosser *et al.*, 2002). Indeed, the use of zebrafish could be effective for identifying safer NSAIDs, but it will be interesting to confirm if hiPSC-CMs can respond better to NSAIDS if COX-2 is constitutively active.

Our results demonstrate the usefulness of the zebrafish model for early detection of cardiotoxic liabilities. But how do these results relate to performances of mammalian models used in the pharmaceutical industry? A pharmaceutical industry survey showed results comparing different animal models and clinical data for 150 compounds (Olson *et al.*, 2000). This report, showed a sensitivity in dogs of approximately 80%. With a similar approach, the Japanese pharmaceutical industry published a survey on 142 drugs (Tamaki *et al.*, 2013), which reported a combined animal sensitivity of 62% for cardiovascular activity. More recently, another pharmaceutical consortium published a survey on 182 drugs (Monticello *et al.*, 2017), in which they report 3% sensitivity and 91% specificity for rodents; 87% sensitivity, and 62% specificity for dogs; and 20% sensitivity and 84% specificity for nonhuman primates (NHP). These results indicate that zebrafish reaches a better sensitivity than rodents and NHP and very similar predictive performance as dogs, which is the standard preclinical regulatory model for addressing cardiotoxicity. In summary, our results suggest that the zebrafish cardiovascular system permits a better prediction of cardiotoxic compounds than traditional hiPSC-CM based models (higher sensitivity). Then, the main risk arising from a lower specificity (55.3%) is the rejection of potential “noncardiovascular risk” candidates. In early drug development phases this pitfall is probably not as relevant as chemical libraries have to be narrowed down to discard the more noxious candidates. In this sense, the use of zebrafish larvae would allow the selection of *bona fide* safe candidates for subsequent phases in which mammalian models are obligatory and would be able to confirm the safety of candidates.

## ETHICAL STUDY APPROVAL

All protocols have been approved by the Institutional Animal Care and Use Ethic Committee at the Barcelona Biomedical Research Park (PRBB–IACUEC) and implemented according to national and European regulations. All experiments were carried out in accordance with the principles of the 3Rs. Specifically, experiments have been performed up to 120 hpf; a developmental stage in which zebrafish embryos are still not considered animals by European laws (Directive 568 2010/63/EU). Hence, all protocols refer to the approved protocol “Zebrafish (Danio 569 rerio) breeding for colony maintenance and transgenic colony creation” (CEA-OH/9421/2).

## SUPPLEMENTARY DATA

Supplementary data are available at *Toxicological Sciences* online.

## DECLARATION OF CONFLICTING INTERESTS

S.D., R.M., M.R.B., C.C., T.P., and J.T. are current employees at ZeClinics. R.G.S. and J.M. are current employees at Chemotargets. ZeCardio and Clarity 2.0.5. are commercial software’s to be used under purchase.

## Supplementary Material

kfz165_Supplementary_Data
