# Supplementary material for: Comparison of Zebrafish Larvae and hiPSC Cardiomyocytes for Predicting Drug-Induced Cardiotoxicity in Humans
Source: Toxicol Sci. 2019 Jul 30;171(2):283–95. doi: 10.1093/toxsci/kfz165 (PMC6760275; doi:10.1093/toxsci/kfz165)
Supplement: kfz165_Supplementary_Data [file kfz165_supplementary_data.zip › toxsci-19-0211-File012.docx]

**Supplementary Material. Dyballa et al, 2019**

**Supplementary Figure 1.** **The HTS setup for ZeCardio^TM^ data acquisition.** The single steps of a routine of sampling, imaging and dispensing a zebrafish larva are shown. The entire hardware arrangement is shown in the center and the single steps are represented by the panels. (A) The LP Sampler^TM^ (right) aspires a larva from the 96 well plate with its aspiration head (left). (B) The larva is flushed through the tubing to the VAST BioImager^TM^ imaging chamber (left). In the thin glass capillary (middle) it is oriented to the desired position in xy and rotated to the desired angle. A screenshot of the image acquired by the VAST onboard camera is shown (right). (C) After successful positioning VAST sends a trigger to the microscope to signal the start of the high resolution imaging sequence. (D) The Autofocus function of the Leica LASX software focuses the heart and the time-lapse movie is acquired. After imaging the microscope signals to the VAST to continue. If more than one position have been defined the larva is oriented to the next position and then imaged again. (E) After all positions have been imaged the larva is returned to its well on a destination plate.

**Supplementary Figure 2. ZeCardio^TM^ software for cardiovascular analyses.** Screenshots from the ZeCardio^TM^ GUI are shown. Video data can be converted imported and organized in ZeCardio. The GUI allows quick access to all video data (heart video and vasculature video panel shown on the top). To generate numerical outputs a heart or vessel selection is made by the user in form of a simple line. For heart analysis (Cardiac Features panel, left hand side) a line is drawn from ventricle to atrium along the heart axis. Perpendicular lines are generated automatically and immediately and the kymographs for each line are displayed underneath the original video. The top kymograph is of the main heart axis. The kymograph in the middle is of the ventricle, note the green ventricle line in the video panel and also in the kymograph panel indicating that the selection is classified as ventricle. The kymograph is segmented for individual beats detection and the segmentation is shown as a red outline. The green curve underneath the ventricle kymograph represents the inverse of the length of the ventricle. This means that peaks in this curve correspond to contractions of the ventricle. A fitted curve is shown in grey. On the bottom is the kymograph of the atrium (blue line in the video panel and in kymograph curve), with blue curve indicating atrial contractions and a fitted curve in grey. Numerical values of the calculations are shown in the GUI immediately upon draw (Cardiovascular features panel, right hand side). Several checkboxes allow flagging specific phenotypes such as AV Coupling Defect, Bigeminy, etc. For vascular analysis (Vascular Features panel) a line selection for vein or artery is drawn by the user. The kymograph of the vessel is shown below (left hand side shows an example for artery, right hand side for vein). The red line underneath the kymograph the represents the average velocities of that time point. Note that for arteries the pulsation of the blood can be well detected by this representation. Below the curve the kymograph and segmentation (red outlines) are shown. Boxplots below indicate the distribution of velocities and the graphs next to them show the histogram of velocities. All the numerical values and flagged phenotypes can then be exported in .csv format.

**Supplementary Table 1.** Table indicating details of the chemical library used in this screen: Drug name, Prestwick number, ATC-code, main molecular target(s), year of withdrawal of compounds withdrawn for cardiotoxicity, Number of FAERs Reports, Major Cardiovascular Event (MCE), Number of MCE Reports, Proportional Reporting Ratio (PRR) and zebrafish NOEC.

**Supplementary Table 2.** z-scores for the zebrafish and hiPSC-CM models

**Supplementary Movie 1**. Videos of different zebrafish hearts under different drug conditions to illustrate the diversity of phenotypic features.
