# Supplementary figures and images for: Comparison of Zebrafish Larvae and hiPSC Cardiomyocytes for Predicting Drug-Induced Cardiotoxicity in Humans
Source: Toxicol Sci. 2019 Jul 30;171(2):283–95. doi: 10.1093/toxsci/kfz165 (PMC6760275; doi:10.1093/toxsci/kfz165)

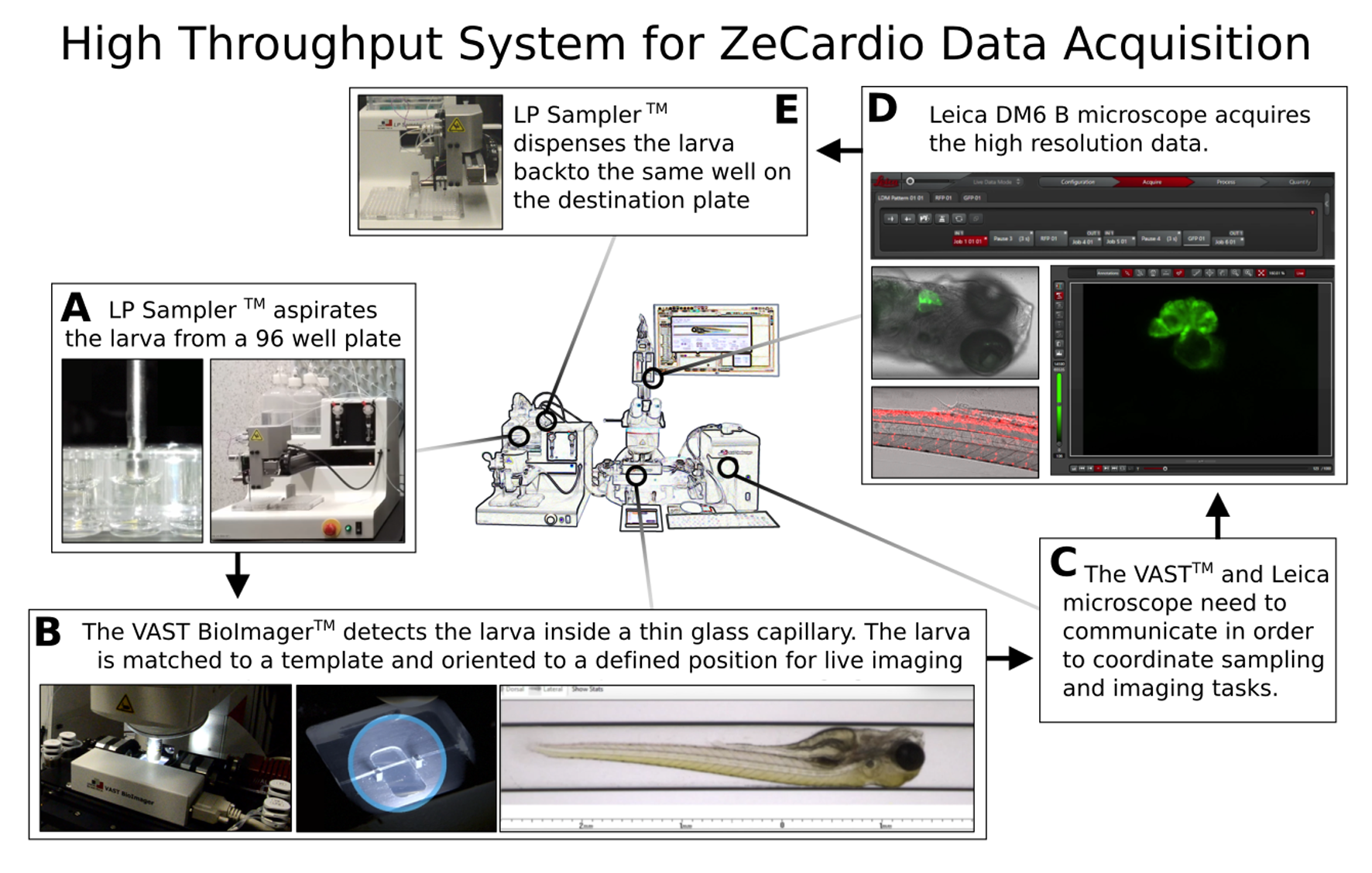

Supplement: kfz165_Supplementary_Data [file kfz165_supplementary_data.zip › toxsci-19-0211-File007.tif]

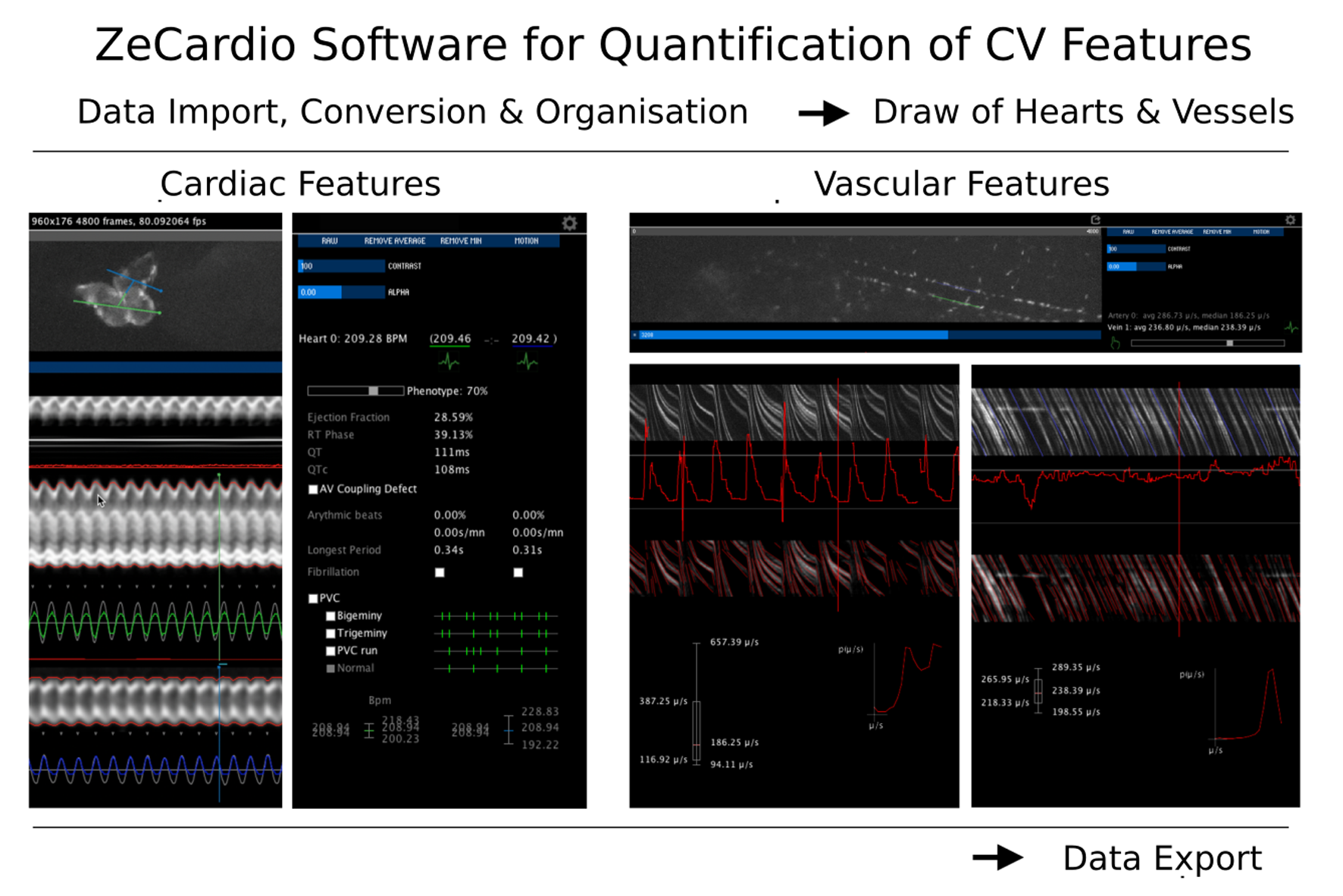

Supplement: kfz165_Supplementary_Data [file kfz165_supplementary_data.zip › toxsci-19-0211-File008.tif]
